# Molecular characterization and differential effects of levofloxacin and ciprofloxacin on the potential for developing quinolone resistance among clinical *Pseudomonas aeruginosa* isolates

**DOI:** 10.3389/fmicb.2023.1209224

**Published:** 2023-09-08

**Authors:** Zeina A. Kanafani, Ahmad Sleiman, Jim Abi Frem, George Doumat, Amal Gharamti, Bassam El Hafi, Michel Doumith, Majed F. AlGhoribi, Souha S. Kanj, George F. Araj, Ghassan M. Matar, Antoine G. Abou Fayad

**Affiliations:** ^1^Division of Infectious Diseases, Department of Internal Medicine, American University of Beirut Medical Center, Beirut, Lebanon; ^2^Center for Infectious Diseases Research, American University of Beirut, Beirut, Lebanon; ^3^Faculty of Medicine, Department of Experimental Pathology, Immunology and Microbiology, American University of Beirut, Beirut, Lebanon; ^4^World Health Organization Collaborating Center for Reference and Research on Bacterial Pathogens, Beirut, Lebanon; ^5^Infectious Diseases Research Department, King Abdullah International Medical Research Center, Riyadh, Saudi Arabia; ^6^King Saud bin Abdulaziz University for Health Sciences, Riyadh, Saudi Arabia; ^7^Department of Pathology and Laboratory Medicine, American University of Beirut Medical Center, Beirut, Lebanon

**Keywords:** *Pseudomonas aeruginosa*, hospital-acquired infection, nosocomial infection, antimicrobial resistance, fluoroquinolone resistance, fitness cost

## Abstract

**Background:**

Fluoroquinolones are some of the most used antimicrobial agents for the treatment of *Pseudomonas aeruginosa*. This study aimed at exploring the differential activity of ciprofloxacin and levofloxacin on the selection of resistance among *P. aeruginosa* isolates at our medical center.

**Methods:**

233 *P. aeruginosa* clinical isolates were included in this study. Antimicrobial susceptibility testing (AST) was done using disk diffusion and broth microdilution assays. Random Amplification of Polymorphic DNA (RAPD) was done to determine the genetic relatedness between the isolates. Induction of resistance against ciprofloxacin and levofloxacin was done on 19 isolates. Fitness cost assay was done on the 38 induced mutants and their parental isolates. Finally, whole genome sequencing was done on 16 induced mutants and their 8 parental isolates.

**Results:**

AST results showed that aztreonam had the highest non-susceptibility. RAPD results identified 18 clusters. The 19 *P. aeruginosa* isolates that were induced against ciprofloxacin and levofloxacin yielded MICs ranging between 16 and 256 μg/mL. Levofloxacin required fewer passages in 10 isolates and the same number of passages in 9 isolates as compared to ciprofloxacin to reach their breakpoints. Fitness cost results showed that 12 and 10 induced mutants against ciprofloxacin and levofloxacin, respectively, had higher fitness cost when compared to their parental isolates. Whole genome sequencing results showed that resistance to ciprofloxacin and levofloxacin in sequenced mutants were mainly associated with alterations in *gyrA*, *gyrB* and *parC* genes.

**Conclusion:**

Understanding resistance patterns and risk factors associated with infections is crucial to decrease the emerging threat of antimicrobial resistance.

## Introduction

*Pseudomonas aeruginosa* is a gram-negative infectious agent notorious for causing many hospital-acquired infections (Kaier et al., 2019). Infections due to *P. aeruginosa* have been increasing worldwide (Rosenthal et al., 2014; Athanasiou and Kopsini, 2018), and they are particularly challenging to treat owing to the intrinsically resistant nature of this bacteria to several commonly used antimicrobial agents. The bacterium itself possesses a cell wall with low permeability, and it easily acquires resistance to commonly used antimicrobial agents (Dantas et al., 2014; Gupta et al., 2016). This pathogen can cause severe infections, such as bacteraemia, pneumonia, bloodstream infections and intraabdominal infections (Folic et al., 2020). Moreover, *P. aeruginosa* is a leading pathogen in ventilator-associated pneumonias (VAPs) globally (Maraolo et al., 2017). Lately, MDR and XDR (multidrug resistant and extensively drug resistant respectively) *P. aeruginosa* constitutes a major health threat especially in endemic areas (Karampatakis et al., 2018). In Lebanon, a recent study showed that *P. aeruginosa* is the second most common agent isolated in VAP (Kanafani et al., 2019).

Fluoroquinolones are one of the most used antibiotic classes for the treatment of *P. aeruginosa*. Fluoroquinolones, particularly ciprofloxacin and levofloxacin, have some beneficial pharmacokinetic characteristics which render them suitable to use. Furthermore, fluoroquinolones are the only orally administered therapy available to treat *P. aeruginosa* infections. Unfortunately, these factors had favored the widespread use of fluoroquinolones for *P. aeruginosa* infection treatment to the extent that they were rendered inefficient in many cases due to emergence of resistance (Yang et al., 2015). Fluoroquinolones resistance in *P. aeruginosa* is primarily attributed to two mechanisms, mutations in the target genes coding enzymes *GyrA*, *ParC*, and *GyrB*, or an increase in the expression of the efflux pumps MexAB-OprM and MexCD-OprJ through mutations in their regulatory genes *mexR* and *nfxB*, respectively (Higgins et al., 2003).

Furthermore, there is a differential effect on the risk of developing resistance between the different classes of fluroquinolones. The literature shows that the use of levofloxacin is associated with increased resistance as compared to ciprofloxacin. One study on clinical isolates of *P. aeruginosa* showed that the use of levofloxacin, but not ciprofloxacin, is a risk factor for isolating fluoroquinolone-resistant strains (Kaye et al., 2006). Another clinical study on isolates from nosocomial infections showed that treating *P. aeruginosa* with levofloxacin is associated with increased rates of fluoroquinolones resistance. However, the use of ciprofloxacin showed no association with resistance (Lee et al., 2010).

On the other hand, an *in vitro* study comparing the development of resistance when using either ciprofloxacin or levofloxacin, found that ciprofloxacin showed a stronger ability to kill the bacteria while rendering it more susceptible to resistance (Zhao et al., 2020).

Fluoroquinolones-resistant *P. aeruginosa* prevalence in Lebanon is significant, representing 22.7% of nosocomial infections in 2013 (Chamoun et al., 2016). The alarming rate at which resistance to fluoroquinolones is emerging in *P. aeruginosa* emphasizes the need to further characterize this resistance pattern. Therefore, the aim of this study is to explore the molecular characteristics of *P. aeruginosa* infections at the American University of Beirut Medical Center and provide at the molecular level the differential activity of ciprofloxacin and levofloxacin on the selection of resistance among *P. aeruginosa* isolates.

## Methodology

### Collection and identification of isolates

A total of 233 *P. aeruginosa* clinical isolates, collected between 2017 and 2019, were included in the study. The patient’s samples were processed, pathogens isolated and identified [using the Matrix-Assisted Laser Desorption/Ionization Time of Flight (MALDI-TOF) system (Bruker Daltonik, GmbH, Bremen, Germany)] with a score of green flags at the Clinical Microbiology Laboratory of the Department of Pathology and Laboratory Medicine (CML-PLM) at the American University of Beirut Medical Center, Beirut, Lebanon, and referred to our laboratory (Marí-Almirall et al., 2017). The isolates were assigned a sequential number upon reception, with repeat samples from the same patient being given letter designations with their original numbers instead of new numbers.

### Disk diffusion

The experiment was performed using the Kirby-Bauer technique. For each isolate, a bacterial suspension equivalent to 0.5 MacFarland was prepared. Then it was subcultured on a round Mueller-Hinton agar plate, in all the directions, to ensure that the bacterial suspension covered all the plate using a sterile swab. The plate was left for around 10 min closed on the bench, followed by the addition of the 8 tested antimicrobials. The plate was then incubated at 37°C for 18–24 h after which the zone of inhibition diameters was measured, and the results were interpreted according to the CLSI M100 guideline (EM100 connect – CLSI M100 ED29, 2019).

### Broth microdilution assay

Broth microdilution was performed with ciprofloxacin and levofloxacin on 19 selected *P. aeruginosa* isolates. Serial dilution took place between columns 1 and 11 to have a concentration ranging from 64 μg/mL to 0.0625 μg/mL. The plate was then placed in the incubator at 37°C for 18 h, and the experiments were run in duplicates for each bacterial isolate. The results were interpreted according to the CLSI M100 guideline (EM100 connect – CLSI M100 ED29, 2019).

### Random amplification of polymorphic DNA (RAPD)

To further characterize our isolate collection beyond phenotypic testing and to assess their genomic relatedness, RAPD was employed using the illustra™ Ready-To-Go RAPD Analysis Beads (GE Healthcare Life Sciences, Marlborough, MA, United States) with the provided Primer 2 (5′-d[GTTTCGCTCC]-3′). A statistically representative selection of isolates from each group was evaluated by RAPD, and the resulting gels were analyzed using BioNumerics 6.6 (Applied Maths NV, Belgium) to generate Unweighted Pair Group Method with Arithmetic mean (UPGMA) dendrograms.

### Induction of resistance

Induction of resistance was done on ciprofloxacin and levofloxacin against 19 selected *P. aeruginosa* clinical isolates. Based on the BMD results of each isolate on ciprofloxacin and levofloxacin, Mueller-Hinton agar plates supplemented with half the MIC of each antibiotic were prepared. Then each isolate was subcultured on agar plates supplemented with its equivalent concentration of each antibiotic separately. The number of passages on each concentration was dependent on the growth rate of the isolate. Once a heavy growth was achieved, an additional passage was done then the isolates were transferred to a 2-fold higher concentration. This was done until each isolate was induced to reach a concentration of 8 and 16 μg/mL of ciprofloxacin and levofloxacin, respectively (Gilbert et al., 2001).

### Fitness cost assay

The tested isolates were first subcultured on LB agar and incubated at 37°C for 18–24 h. The next day, a loop full of each bacterial isolate was transferred into 10 mL of sterile cation adjusted Mueller-Hinton broth and incubated at 37°C for 18–24 h. Then, the turbid inoculated broth of each isolate was diluted at a 1:1000 ratio. The latter was then transferred into 4 distinct wells (200 μL each) of a 96 well microtiter plate. The replication rate of each tested isolate was measured using a plate reader (OD 600 nm) for 16 h with reads at 30 min intervals. The results were then averaged, normalized, and plotted on a graph (Mu et al., 2016).

### Phe-Arg-β-naphthylamide inhibitor assay

Following the induction of all the 19 isolates against ciprofloxacin and levofloxacin, their MICs were determined for both antimicrobial agents with and without a multi-drug efflux pump inhibitor Phe-Arg-β-naphthylamide (PAβN). Ciprofloxacin or levofloxacin-PAβN inhibitor combinations experiment was performed by adding fixed concentrations of the inhibitors to the experimental wells of a standard antimicrobial broth microdilution assay. We followed CLSI guidelines in this assay. However, minor modifications to broth volumes were made in order to accommodate the presence of the PAβN inhibitor while keeping the concentrations of the ciprofloxacin or levofloxacin and the bacterial suspensions in accordance with CLSI recommendations. For each isolate, PAβN inhibitor was used at a fixed concentration of 25 μg/mL (Schuster et al., 2019). The MICs of the tested isolates were interpreted according to the CLSI M100 guideline (EM100 connect – CLSI M100 ED29, 2019).

### Whole genome sequencing (WGS)

Genomic DNA of mutant and parental strains was extracted from their overnight cultures with the QIAamp DNA Mini Kit (Qiagen, Hilden, Germany) and then prepared for sequencing using the Nextera XT DNA library preparation kit (Illumina, Cambridge, United Kingdom) according to the manufacturer’s protocols. Sequencing was performed on the Illumina MiSeq instrument using the 2×300 paired-end mode at the microbial genomic facilities in the Infectious Diseases Research Department, King Abdullah International Medical Research Center, Riyadh Saudi Arabia.

### Bioinformatics analysis of the isolates

Reads thus generated were first filtered to remove low quality reads with Trimmomatic 0.38 (Bolger et al., 2014)^1^ and then assembled using Spades 3.13.0 (Bankevich et al., 2012)^2^ with the default options. *In silico* multilocus sequence typing was determined with the mlst 2.18.0 software^3^ using the allelic sequences and ST profiles from the *P. aeruginosa* public MLST database.^4^ The alterations in genes associated with resistance to ciprofloxacin and levofloxacin were initially identified by BLAST^5^ and then confirmed by mapping with Genefinder^6^ using the sequence of strain PAO-1 (NC_002516.2) as reference.

## Results

### Antimicrobial susceptibility testing

The disk diffusion results showed that out of the 233 *P. aeruginosa* isolates, 87 (37%) were non-susceptible to aztreonam, 63 (27%) were non-susceptible to imipenem, 58 (25%) were non-susceptible to Piperacillin-tazobactam, 55 (24%) were non-susceptible to ciprofloxacin, 45 (19%) were non-susceptible to ceftazidime, 32 (14%) were non-susceptible to cefepime, 28 (12%) were non-susceptible to amikacin, and 25 (11%) were non-susceptible to gentamicin (Table 1).

**Table 1:** Antimicrobial susceptibility testing results of all the 233 *P. aeruginosa* isolates.

Antimicrobial Agents	No. Non-Susceptible (%)
Ceftazidime	45 (19%)
Cefepime	32 (14%)
Imipenem	63 (27%)
Aztreonam	87 (37%)
Ciprofloxacin	55 (24%)
Amikacin	28 (12%)
Gentamicin	25 (11%)
Piperacillin-tazobactam	58 (25%)

**Figure 1:**
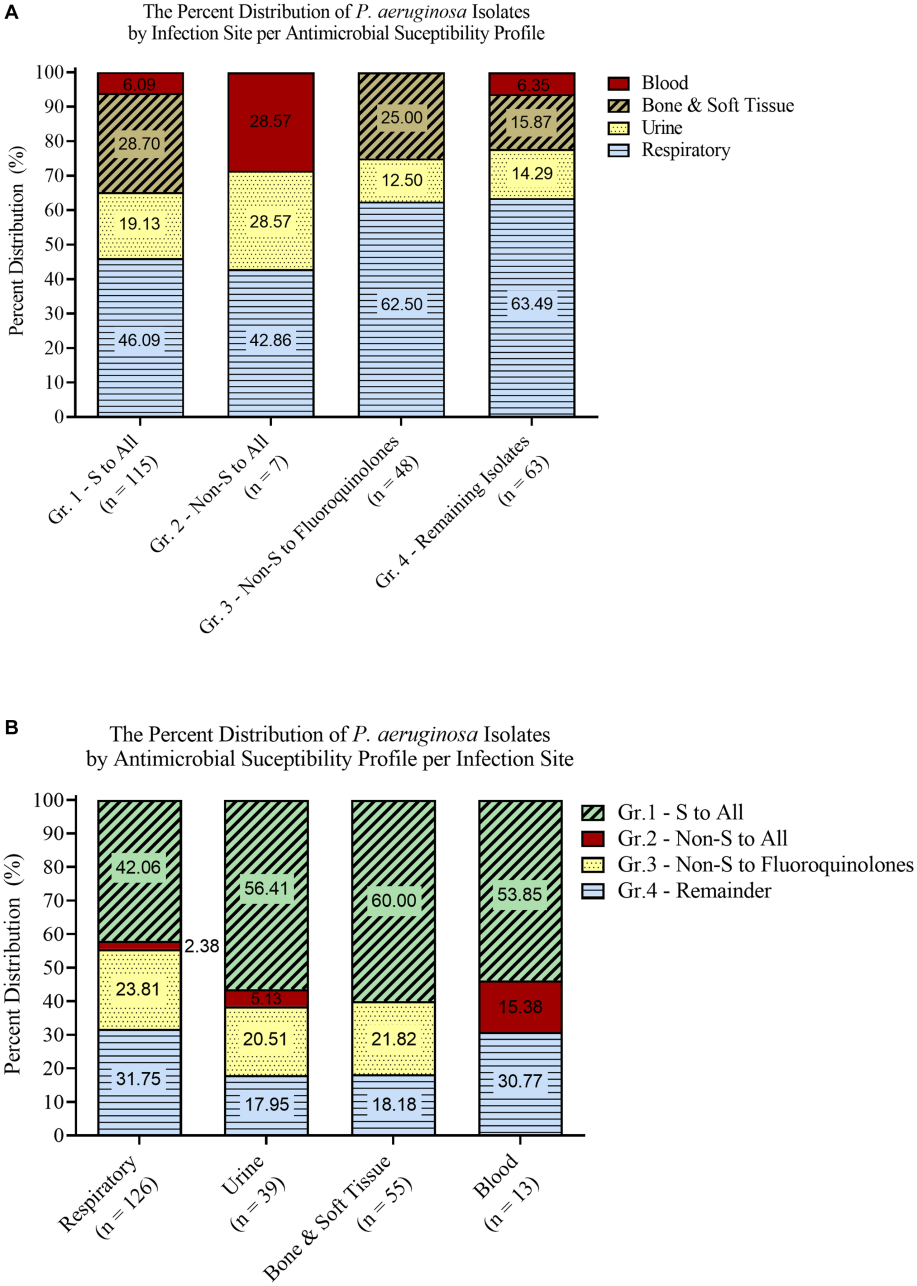
Isolate grouping stratified according to infection site (A) and antimicrobial susceptibility profiles (B).

Based on their infection site and susceptibility profiles, the isolates were stratified into different grouping schemes. The first scheme grouped the isolates based on their sampling site into Marí-Almirall et al. (2017), Respiratory (Athanasiou and Kopsini, 2018), Urine (Rosenthal et al., 2014), Bone and Soft Tissue (Gupta et al., 2016), and Blood (Figure 1A). A second separate scheme grouped the isolates based on their antimicrobial susceptibility profiles into Marí-Almirall et al. (2017), Susceptible to all, (Athanasiou and Kopsini, 2018), Non-Susceptible to all (Rosenthal et al., 2014), Non-Susceptible to Fluoroquinolones (Gupta et al., 2016), and Remaining isolates (Figure 1B). Within the second scheme, the isolates did not overlap across different groups.

### Molecular epidemiology

Upon assessing the overall genomic relatedness among the analyzed isolates with an 85% genomic similarity cutoff, a total of 18 clusters were identified (Supplementary Figure S1). There were three major clusters with >20 isolates each, four intermediate clusters with 7–18 isolates each, and 11 minor clusters with each containing 2–4 isolates.

According to their sites of infection, when breaking down the isolates into their respective groups, 19 clusters were observed. The respiratory group contained nine clusters (Supplementary Figure S2): 2 major clusters with >20 isolates each, two intermediate clusters with 9 and 17 isolates respectively, and five minor clusters with 2–5 isolates each. The urine group contained 5 clusters (Supplementary Figure S3) with 2–6 isolates each. The bone & soft tissue group contained 4 clusters (Supplementary Figure S4): 2 intermediately sized with 7 and 11 isolates respectively, and two minor clusters with 2 and 5 isolates, respectively. Finally, the blood group only contained one cluster comprised of 5 isolates (Supplementary Figure S5).

On the other hand, when breaking down the overall dendrogram into groups based on the isolates’ antimicrobial susceptibility profiles, a total of 18 clusters were observed. Group 1 – “Susceptible to all” contained 6 clusters (Supplementary Figure S6): one major cluster with 20 isolates, three intermediate clusters with 9–15 isolates each, and two minor clusters with 2 and 4 isolates, respectively. Group 2 – “Non-Susceptible to all” did not contain any cluster (Supplementary Figure S7). Group 3 – “Non-Susceptible to Fluoroquinolones” contained seven clusters of 2–8 isolates each (Supplementary Figure S8). Finally, group 4 – “Remaining isolates” contained five clusters (Supplementary Figure S9): 1 cluster with 19 isolates, one cluster with 11 isolates and three clusters with 2–5 isolates each. Furthermore, wgMLST was performed on 8 isolates that represent the major clusters identified (Supplementary Figure S14).

### Induction of resistance

Out of the 233 *P. aeruginosa* isolates, 19 were selected for the induction of resistance. The presence of distinct clusters provided guidance in selecting appropriate isolates for downstream applications. Therefore, the selection criteria were based on the susceptibility profiles, site of infection, and the clusters that the isolates belonged to based on the RAPD results. The common criteria between all the selected isolates were their susceptibility to ciprofloxacin and levofloxacin. The antimicrobial susceptibility testing results showed that the acquisition of ciprofloxacin resistance in *P. aeruginosa* led to levofloxacin resistance too and vice versa. All the 19 *P. aeruginosa* isolates that were induced against levofloxacin became non-susceptible to ciprofloxacin (1 intermediate and 18 resistant). Whereas 18 (95%) out of the 19 *P. aeruginosa* isolates that were induced against ciprofloxacin became non-susceptible to levofloxacin. Before the induction of resistance, the range of the minimal inhibitory concentration (MIC) of the 19 *P. aeruginosa* isolates was between <0.0625 μg/mL and 0.25 μg/mL for ciprofloxacin, and between 0.25 μg/mL and 0.5 μg/mL for levofloxacin. However, after the induction of resistance, the 19 *P. aeruginosa* isolates that were induced against ciprofloxacin yielded MICs ranging between 16 μg/mL and 256 μg/mL for ciprofloxacin and between 1 μg/mL and > 512 μg/mL for levofloxacin. Whereas the 19 *P. aeruginosa* isolates that were induced against levofloxacin yielded MICs ranging between 16 μg/mL and 256 μg/mL for levofloxacin and between 1 μg/mL and 32 μg/mL for ciprofloxacin (Table 2). The median MIC when induced against ciprofloxacin increased from 0.125 to 32 (256 folds) for ciprofloxacin and from 0.5 to 64 (128 folds) for levofloxacin. The median MIC when induced with levofloxacin increased from 0.5 to 32 (64 folds) for levofloxacin and 0.125 to 16 (128 folds) for ciprofloxacin.

**Table 2:** Broth microdilution results of the 19 *P. aeruginosa* isolates against ciprofloxacin and levofloxacin before and after induction of resistance.

			MIC (μg/mL)		
Isolates codes	Before induction		After induction		# of passages*
	Antimicrobial IG	MIC (μg/mL)	Ciprofloxacin	Levofloxacin	
PSA-154	Ciprofloxacin	0.125	256	>512	4
	Levofloxacin	0.5	32	256	3
PSA-021	Ciprofloxacin	<0.0625	32	1	5
	Levofloxacin	0.25	16	32	4
PSA-173b	Ciprofloxacin	0.25	64	64	3
	Levofloxacin	0.5	16	64	3
PSA-173a	Ciprofloxacin	0.25	32	64	3
	Levofloxacin	0.5	16	32	3
PSA-115	Ciprofloxacin	0.125	32	64	4
	Levofloxacin	0.5	8	32	3
PSA-276	Ciprofloxacin	0.125	16	16	4
	Levofloxacin	0.5	32	256	3
PSA-242	Ciprofloxacin	0.25	16	16	3
	Levofloxacin	0.5	8	64	3
PSA-025	Ciprofloxacin	0.125	256	512	4
	Levofloxacin	0.5	4	32	3
PSA-026	Ciprofloxacin	0.25	32	64	3
	Levofloxacin	0.5	16	64	3
PSA-186	Ciprofloxacin	0.125	32	128	4
	Levofloxacin	0.5	32	128	3
PSA-118a	Ciprofloxacin	0.25	128	>512	3
	Levofloxacin	0.5	32	128	3
PSA-192	Ciprofloxacin	0.125	16	32	4
	Levofloxacin	0.25	2	32	4
PSA-182	Ciprofloxacin	0.125	16	32	4
	Levofloxacin	0.5	1	16	3
PSA-058	Ciprofloxacin	0.125	32	32	4
	Levofloxacin	0.5	8	32	3
PSA-285b	Ciprofloxacin	0.125	32	64	4
	Levofloxacin	0.5	4	32	3
PSA-113a	Ciprofloxacin	0.25	128	128	3
	Levofloxacin	0.5	16	64	3
PSA-124	Ciprofloxacin	0.25	16	16	3
	Levofloxacin	0.5	16	16	3
PSA-092	Ciprofloxacin	0.125	64	128	4
	Levofloxacin	0.25	8	16	4
PSA-146b	Ciprofloxacin	0.125	32	64	4
	Levofloxacin	0.5	16	32	3

IG, induced against; *, each passage represents a 2-fold increase in the MIC and the numbers indicate the passages required to reach each antibiotic breakpoint.

In addition to that, when broth microdilution was done on the 19 *P. aeruginosa* isolates that were induced against ciprofloxacin with the addition of the multi-drug efflux pump inhibitor PAβN, no difference in MICs was detected in 2 (10%) isolates, two-fold decrease in 11 (58%) isolates, fourfold decrease in 2 (10%) isolates, and eight-fold decrease in 4 (22%) isolates were detected. However, the results of the 19 *P. aeruginosa* isolates that were induced against levofloxacin showed no difference in MICs in 1 (5%) isolate, two-fold decrease in 11 (58%) isolates, fourfold decrease in 4 (22%) isolates, eight-fold decrease in 2 (10%) isolates, and 16-fold in a decrease in 1 (5%) isolate (Supplementary Table S1).

Moreover, throughout the induction of resistance on the 19 selected *P. aeruginosa* isolates, 17 (89%) isolates grew at a higher rate in plates supplemented with ciprofloxacin compared to that with levofloxacin and only 2 (11%) isolates grew at the same rate. Furthermore, the number of passages needed to reach the antibiotic resistance breakpoint was higher for ciprofloxacin in 10 isolates and equal for ciprofloxacin and levofloxacin in 9 isolates.

### Fitness cost

**Figure 2:**
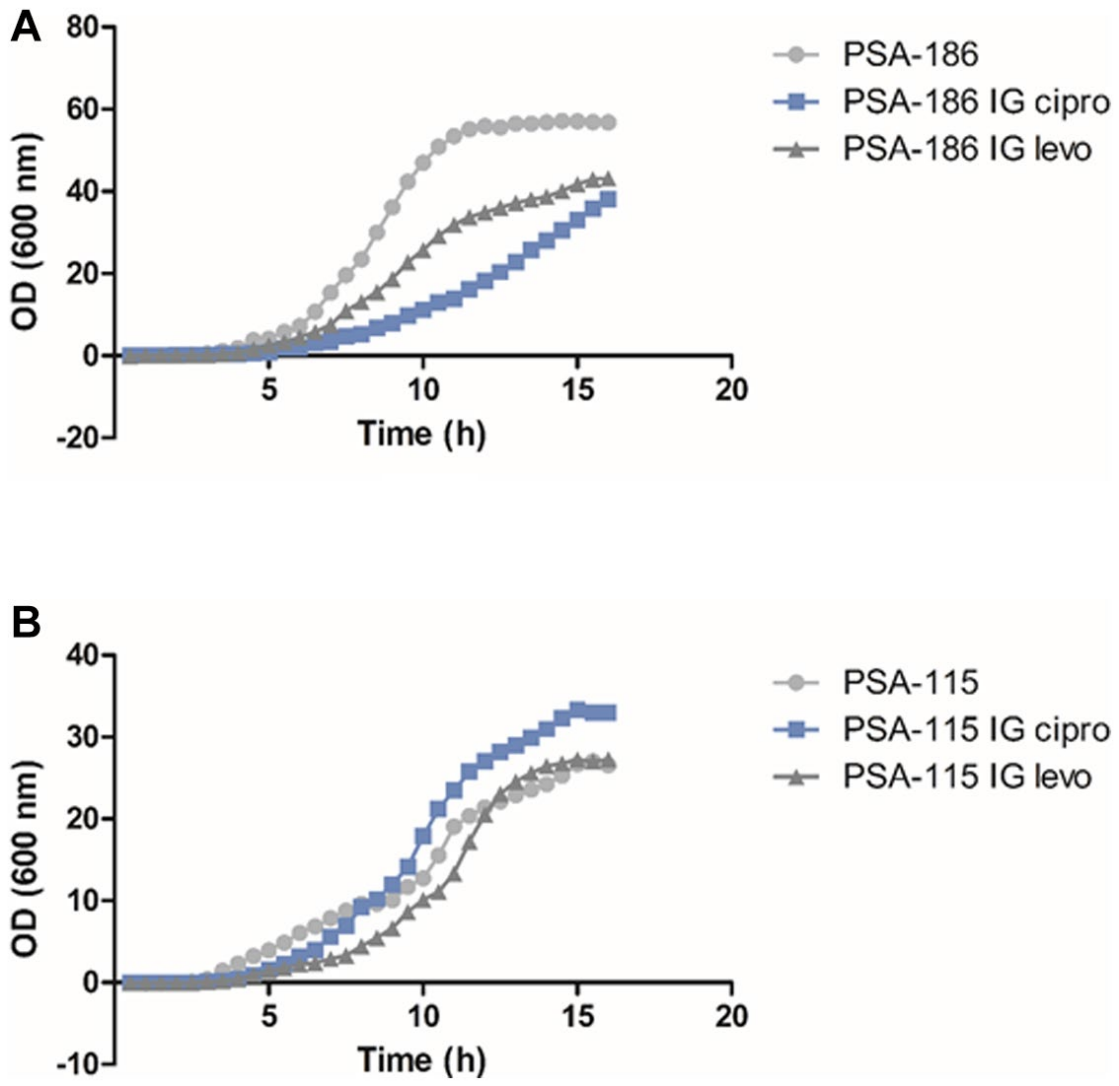
Fitness cost assay results of isolates PSA-115 (B) and PSA-186 (A) compared to their induced mutants. IG cipro, induced against ciprofloxacin; IG levo, induced against levofloxacin.

This experiment was done to evaluate the cost of acquiring fluoroquinolone resistance on the fitness of *P. aeruginosa*. For each isolate, we compared the induced mutants against ciprofloxacin and levofloxacin separately to the parental isolate prior to induction. For ciprofloxacin, 12 (63%) induced isolates showed a higher fitness cost, lower growth rate, with *p*-values ranging between *p* < 0.0001 and 0.0332 when compared to the parental isolates before induction (Figure 2A; Supplementary Figures S10, S11, S13B–D). However, 7 (37%) induced isolates showed no significant change in fitness cost, same growth rate, when compared to their parental isolates prior to induction with p-values ranging between 0.0827 and 0.9252 (Figure 2B; Supplementary Figures S12, S13A). For levofloxacin, 10 (53%) induced isolates showed a higher fitness cost, lower growth rate, with p-values ranging between p < 0.0001 and 0.0391 when compared to the parental isolates before induction (Figure 2A; Supplementary Figures S10, S11, S13A). Whereas 9 (47%) induced isolates showed no significant change in fitness cost, same growth rate, when compared to their parental isolates prior to induction with p-values ranging between 0.0937 and 0.8686 (Figure 2B; Supplementary Figures S12, S13B–D).

### Whole-genome sequencing

Genome sequence analyses confirmed that parental and isogenic mutant isolates belonged to the same sequence types, and which comprised ST244 (*n* = 2), ST357 (*n* = 1), ST253 (*n* = 1), ST308 (*n* = 1), ST664 (*n* = 1), ST810 (*n* = 1) and ST3137 (*n* = 1). Resistance to ciprofloxacin and levofloxacin in sequenced mutants were mainly associated with alterations in *GyrA*, *GyrB* and *ParC*. Characteristically, mutants with a high level of resistance to ciprofloxacin and levofloxacin (i.e., MICs ≥256 μg/mL) had changes in both *GyrA* (Thr83 → Ala/Ile and/or Asp87 → Tyr) and *ParC* (Ser87 → Leu/Trp). Isolate PSA-154 that was induced against ciprofloxacin yielded MICs of 256 μg/mL for ciprofloxacin and > 512 μg/mL for levofloxacin. This high level of resistance was caused by two mutations in the *gyrA* gene (Thr83 → Ala and Asp87 → Tyr) and one in the *parC* gene (Ser87 → Trp). Moreover, another isolates (PSA-025) that was also induced against ciprofloxacin yielded MICs of 256 μg/mL for ciprofloxacin and 512 μg/mL for levofloxacin. Two mutations were detected in these isolates, one in the *gyrA* gene (Thr83 → Ile) and the second was in the *parC* gene (Ser87 → Leu). In comparison, those exhibiting ciprofloxacin MICs between 16 μg/mL and 128 μg/mL or had MIC values ranging between 64 μg/mL and 256 μg/mL for levofloxacin carried only single alterations in *GyrA*. On the other hand, amino acid changes, deletions and/or insertions at positions 466, 467, 468, 476, 477,478, 481, 486, 702, 704, and/or 749 in *GyrB* were detected in mutants with ciprofloxacin and levofloxacin MICs ranging from 4 μg/mL to 32 μg/mL and 32 μg/mL to 64 μg/mL, respectively. A single mutation in the *gyrB* (Ser466 → Phe) of isolate PSA-021 that was induced against ciprofloxacin was detected. Interestingly, this isolate became resistant to ciprofloxacin (MIC: 32 μg/mL) and remained susceptible to levofloxacin (MIC: 1 μg/mL). Only one mutant (PSA-242 IG Cipro) with an MIC of 16 μg/mL against both ciprofloxacin and levofloxacin lacked any modifications in the DNA gyrase or topoisomerase subunits (Table 3).

**Table 3:** Whole genome sequencing results of the 16 induced mutants and their 8 parental isolates.

Isolates	ST	*gyrA*		*gyrB*		*parC*		*mexR*		*nfxB*	
		nuc	prot	nuc	prot	nuc	prot	nuc	prot	nuc	prot
PSA-154	308										
PSA-154-IG cipro	308	247 A > G 259 G > T	T83 > A D87 > Y			260\u00B0C > G	S87W				
PSA-154-IG levo	308	259 G > T	D87 > Y								
PSA-021	664										
PSA-021-IG cipro	664			1,397\u00B0C > T	S466 > F			77 ins T	Frame shift		
PSA-021-IG levo	664			2,245\u00B0C > T	P749 > S					271–272 ins C	Frame shift
PSA-115	3,137										
PSA-115-IG cipro	3,137			1,395–1,404 del CTCCCAGGA	S466-E468 del						
PSA-115-IG levo	3,137			1,425–1,436 del CCTGGGCTGTGG	L476 > I C478-G481 del						
PSA-242	810										
PSA-242-IG cipro	810										
PSA-242-IG levo	810			1,395–1,401 del CTCCCA 2108–2010 del TGG	S466-Q467 del L702 del E704 > Q			271–282 del GACCAGCGCAGC	S88-R91del		deletion
PSA-025	253										
PSA-025-IG cipro	253	248\u00B0C > T	T83 > I			260\u00B0C > T	S87L			182–216 del TACTGAACCAGATCATCCAGGCCTGCGACCTGGAG	Frame shift
PSA-025-IG levo	253			1,459–1,460 ins ACA	N486 ins			353-end del	E118-end del		
PSA-026	244										
PSA-026-IG cipro	244			2,245\u00B0C > T	P749 > S					544 T > C	S185 > P
PSA-026-IG levo	244	259 G > A	D87 > N					374–388 del TCACCCCGGAGGAAC	Frame shift		
PSA-285b	244										
PSA-285b IG cipro	244	259 G > T	D87 > Y							537–547 del ACGGCGCTTCC	Frame shift
PSA-285b-IG levo	244			1,334–1,335 ins TGT	C477 ins			260–261 ins GCAAC	Frame shift		
PSA-113a	357										
PSA-113a-IG cipro	357	248\u00B0C > T	T83 > I							514–515 del TA	Frame shift
PSA-113a-IG levo	357	247 A > G	T83 > A	2,246\u00B0C > A	P749 > H			235–244 del AAACCTGGTC	Frame shift		

ST, sequence type; IG, induced against; nuc, nucleotide; prot: protein.

Genome sequences have also shown that the MexCD,-OprJ efflux regulatory genes *nfxB* and *mexR* were inactivated in 11 (69%) out of the 16 sequenced mutants through various alterations in their respective coding sequences. These alterations ranged from single nucleotide substitutions to deletions or insertions of 1 to 91 nucleotides. Interestingly, the inactivation of *MexR* was identified mainly in mutants induced with levofloxacin, whereas the disruption of NfxR was seen more often in mutants induced with ciprofloxacin (Table 3). However, all the mutants carting these alterations had at most four folds decrease in their respective MICs in the presence of PAβN. Oddly, all mutants generated from strains PSA-154 and PSA-115 that showed the highest MIC shifts in the presence of PAβN (8-to-16-fold decrease) did not show any alterations in these two regulators.

Fitness cost results showed that PSA-154 and PSA-025 that were induced against ciprofloxacin had no significant change in the fitness cost, same growth rate, when compared to their parental isolates. Even though PSA-154-IG ciprofloxacin had 2 mutations in the *gyrA* gene (Thr83 → Ala and Asp87 → Tyr) and one mutation in the *parC* gene (Ser87 → Trp), and PSA-025-IG ciprofloxacin had one mutation in the *gyrA* (Thr83 → Ile) and another one in the *parC* gene (Ser87 → Leu). Moreover, out of the 5 induced mutants that had a single mutation in the *gyrA* gene, 3 (60%) had a higher fitness cost and 2 (40%) had no significant change in the fitness cost when compared to their parental isolates. Furthermore, out of the 9 induced mutants that had alteration in the *gyrB* gene, 6 (66.67%) had a higher fitness cost and 3 (33.33%) had no significant change in the fitness cost when compared to their parental isolates. In addition to that, out of the 11 induced mutants that had the MexCD-OprJ efflux regulatory genes *nfxB* and *mexR* inactivated, 8 (73%) had a higher fitness cost and 3 (27%) had no significant change in the fitness cost when compared to their parental isolates.

## Discussion

*P. aeruginosa* accounts for 10% of all hospital-acquired infections, which makes it the fourth most commonly isolated nosocomial pathogen (Afshari et al., 2012). Yet, the increase in antimicrobial resistance has been an ongoing struggle for a long time, shrinking the repertoire of options to treat these infections. A study assessing *P. aeruginosa* isolates from ICU patients showed that 47.7% of the isolates were drug-resistant, 50% were multidrug resistant, and 2.3% were extensively drug resistant (Gill et al., 2016). Fluoroquinolones are a class of first-line antimicrobials that exhibit anti-pseudomonal activity and are frequently used to treat such infections. In this study, we observe an alarming non-susceptibility of 24% to ciprofloxacin among the *P. aeruginosa* clinical isolates, which is similar to the percentage reported from nosocomial infections in Lebanon (22.7%) (Chamoun et al., 2016). Moreover, a recent study in Lebanon showed that 27% of *P. aeruginosa* isolated from hospitalized patients and 28.6% from ICU patients are resistant to ciprofloxacin (Al-orphaly et al., 2021). This level of resistance is also very close to the percentage reported in a recent national report that investigated the antimicrobial susceptibility profiles of multiple organisms to a number of routinely screened antimicrobial agents across several tertiary care centers (Moghnieh et al., 2019). In fact, all the tested antimicrobials in this study nearly mirrors the national numbers in terms of *P. aeruginosa* susceptibility percentages (Moghnieh et al., 2019) despite the fact that the samples included in this study were exclusively isolated from admitted patients. Therefore, RAPD was utilized to better elucidate the characteristics of the isolate collection and to clarify whether common strains circulate among in-patients or whether they are largely infected by community strains. Following the assessment of the overall genomic relatedness among the analyzed isolates, it appears as though there is not a specific criterium that governed their distribution across their sites of infection as well as their antimicrobial susceptibility profiles. The majority of detected clusters include samples from different infection sites and susceptibility profiles. However, most isolates from respiratory samples, half of the isolates from urine, and two-third of the isolates from bone and soft tissue, seem to group into distinct clusters which signifies the possibility of hospital spread of isolates circulating between different patients. On the contrary, samples from bloodstream infections are largely independent since there was only 1 cluster with lower overall genomic similarity between the isolates than observed elsewhere. On the other hand, when the samples were analyzed based on their antimicrobial susceptibility grouping, we observed multiple clusters in each group. This shows that common isolates sharing similar antimicrobial susceptibility profiles could be infecting multiple patients at different sites. These findings show that despite our *P. aeruginosa* clinical samples sharing a very similar antimicrobial susceptibility profile with the larger community registry, there is a number of common strains that seem to circulate among in-patients within and at different sites of infection.

The main goal behind the induction of resistance was to mimic the idea of treating patients infected with fluoroquinolone susceptible *P. aeruginosa* with either ciprofloxacin or levofloxacin. Our data showed that exposure to either ciprofloxacin or levofloxacin will lead to resistance against both classes of fluoroquinolones, in contrast to the results from clinical studies (Kaye et al., 2006; Lee et al., 2010; Zhao et al., 2020). One study showed that the risk of isolating a resistant *P. aeruginosa* increased with exposure to levofloxacin, however it did not change with exposure to ciprofloxacin (Kaye et al., 2006). Another study assessing the rate of *P. aeruginosa* resistance among nosocomial infections showed that levofloxacin, administered either orally and parenterally, was positively correlated with developing resistance to either levofloxacin or ciprofloxacin (Lee et al., 2010), whereas treatment with ciprofloxacin was not correlated with resistance to fluoroquinolones (Lee et al., 2010). Nonetheless, an *in vitro* study showed results similar to our study, contrary to the clinical studies. It was shown that ciprofloxacin had a higher ability to kill *P. aeruginosa* but it also showed more susceptibility to resistance (Zhao et al., 2020).

Another important parameter in characterizing resistance is the frequency and speed of developing resistance, reflected by the number of passages needed to reach the resistance breakpoint. Our results showed that levofloxacin required fewer number of passages in a total of 10 tested isolates and an equal number of passages in a total of 9 tested isolates when compared to ciprofloxacin. Thus, resistance against levofloxacin developed faster than ciprofloxacin.

Moreover, the growth rate data showed that fluroquinolone resistance in general reduced the growth rate of the organism, whereby ciprofloxacin resistance impacting the growth rate of the isolates more compared to levofloxacin resistance. A study assessing cost of fitness on quinolone resistance showed that low level mutations showed no cost on fitness in more than half of the organisms. Conversely, organisms with high level mutations showed decreased fitness in all isolates. Interestingly, after serial passage in the laboratory medium, mutant fitness increased again by compensatory mutations (Kugelberg et al., 2005).

To summarize, treatment with ciprofloxacin induces more resistance, while treatment with levofloxacin reaches resistance breakpoint faster and affected the growth rate.

At the molecular level, whole genome sequencing results showed that resistance to ciprofloxacin and levofloxacin in sequenced mutants were mainly associated with alterations in *gyrA*, *gyrB*, and *parC*. Interestingly, the results we got from the induced mutants are similar to the results in studies tackling fluoroquinolone resistance in *P. aeruginosa* clinical isolates. Nouri et al. examined mutations in *gyrA* and *parC* genes in 64 fluoroquinolone resistant *P. aeruginosa* clinical isolates (Nouria et al., 2016). Their results showed that a single *gyrA* substitution (Thr-83 → Ile) was linked with MIC values ranging between 4 and 64 μg/mL for ciprofloxacin and between 4 and 32 μg/mL for levofloxacin. Moreover, isolates with a single *gyrA* substitution (Thr-83 → Ile) and a single *parC* substitution (Ala-88 → Pro or Ser-87 → Leu) had MICs ranging between 8 and 128 or 16 and 256 μg/mL for ciprofloxacin and ranging between 8 and 64 or 8 and 256 μg/mL for levofloxacin. In addition to that, isolates with double *gyrA* substitutions (Thr-83 → Ile and Asp-87 → Asn) and single *parC* substitutions (Ser-87 → Leu) had MICs ranging between 32 and 256 μg/mL for both ciprofloxacin and levofloxacin. Their results showed that three coexisting mutations in *gyrA* and *parC* are associated with higher MIC values for both ciprofloxacin and levofloxacin as compared to two coexisting mutations in *gyrA* and *parC*. Moreover, the latter was associated with higher MIC values for both antimicrobial agents when compared to single mutations in *gyrA* (Nouria et al., 2016). Moreover, Yang et al. work revealed that in 65 ciprofloxacin resistant *P. aeruginosa* clinical isolates a missense mutation in *gyrA* gene (Thr-83 → Ile) was the most detected, followed by missense mutation in *parC* (Ser-87 → Leu), and independent missense mutations in *gyrB* (Ser-467 → Phe and Gln-468 → His). They also showed that *P. aeruginosa* isolates with double mutations (*gyrA* and *gyrB* or *gyrA* and *parC*) had higher MIC values against ciprofloxacin compared to the ones with single *gyrA* mutations (Yang et al., 2015).

The differential effects of ciprofloxacin and levofloxacin on the emerging resistance against *P. aeruginosa* should be taken into consideration when devising a treatment plan. Factors such as the extent of the resistance, speed of resistance, and cost of fitness will affect the clinical decision making when translated into everyday practice.

Understanding the mechanism of action driving the resistance against quinolones can provide insight into developing a new generation of therapeutic drugs that can overcome the resistance.

Further studies assessing the characteristics of resistance in a clinical setting are needed to complete the picture and guide therapy.

## Data availability statement

The datasets presented in this study can be found in online repositories. The names of the repository/repositories and accession number(s) can be found in the article/Supplementary material.

## Author contributions

ZK, AS, BE, MD, and MA executed the experiments and wrote the manuscript. ZK, SK, JF, GD, and AG performed the clinical analysis. AA, ZK, and GM designed the experiments and planned the study. AA, ZK, SK, and GM proof read and edited the overall article. All authors contributed to the article and approved the submitted version.

## Funding

This work was funded by the Medical Practice Plan (MPP) grant from AUBMC.

## Conflict of interest

The authors declare that the research was conducted in the absence of any commercial or financial relationships that could be construed as a potential conflict of interest.

## Publisher’s note

All claims expressed in this article are solely those of the authors and do not necessarily represent those of their affiliated organizations, or those of the publisher, the editors and the reviewers. Any product that may be evaluated in this article, or claim that may be made by its manufacturer, is not guaranteed or endorsed by the publisher.

## Footnotes

^1^ http://www.usadellab.org/cms/index.php?page=trimmomatic

^2^ http://cab.spbu.ru/software/spades

^3^Seemann T., mlst Github, https://github.com/tseemann/mlst.

^4^ https://pubmlst.org/organisms/pseudomonas-aeruginosa

^5^ https://blast.ncbi.nlm.nih.gov/Blast.cgi

^6^Github, https://github.com/ukhsa-collaboration/gene_finder.
