# Supplementary material for: Molecular characterization and differential effects of levofloxacin and ciprofloxacin on the potential for developing quinolone resistance among clinical Pseudomonas aeruginosa isolates
Source: Front Microbiol. 2023 Sep 8;14:1209224. doi: 10.3389/fmicb.2023.1209224 (PMC10514475; doi:10.3389/fmicb.2023.1209224)
Supplement: Supplementary file 1 [file Data_Sheet_1.pdf]

Supplementary Material

Supplementary Tables

Table S1: Broth microdilution results of the 19 *P. aeruginosa* isolates against ciprofloxacin and levofloxacin before and after induction of resistance (with and without multi-drug efflux pump inhibitor PAβN)

Isolates codes	Antibiotics	MIC (µg/mL)		
		Before induction	After induction	
			Without PAβN	With PAβN
PSA-154	Ciprofloxacin	0.125	256	32
	Levofloxacin	0.5	256	16
PSA-021	Ciprofloxacin	<0.0625	32	16
	Levofloxacin	0.25	32	16
PSA-173b	Ciprofloxacin	0.25	64	8
	Levofloxacin	0.5	64	16
PSA-173a	Ciprofloxacin	0.25	32	32
	Levofloxacin	0.5	32	32
PSA-115	Ciprofloxacin	0.125	32	4
	Levofloxacin	0.5	32	4
PSA-276	Ciprofloxacin	0.125	16	8
	Levofloxacin	0.5	256	32
PSA-242	Ciprofloxacin	0.25	16	8
	Levofloxacin	0.5	64	32
PSA-025	Ciprofloxacin	0.125	256	128
	Levofloxacin	0.5	32	16
PSA-026	Ciprofloxacin	0.25	32	16
	Levofloxacin	0.5	64	32
PSA-186	Ciprofloxacin	0.125	32	16
	Levofloxacin	0.5	128	64
PSA-118a	Ciprofloxacin	0.25	128	64
	Levofloxacin	0.5	128	64

PSA-192	Ciprofloxacin	0.125	16	8
	Levofloxacin	0.25	32	16
PSA-182	Ciprofloxacin	0.125	16	4
	Levofloxacin	0.5	16	4
PSA-058	Ciprofloxacin	0.125	32	16
	Levofloxacin	0.5	32	16
PSA-285b	Ciprofloxacin	0.125	32	8
	Levofloxacin	0.5	32	16
PSA-113a	Ciprofloxacin	0.25	128	64
	Levofloxacin	0.5	64	16
PSA-124	Ciprofloxacin	0.25	16	16
	Levofloxacin	0.5	16	8
PSA-092	Ciprofloxacin	0.125	64	8
	Levofloxacin	0.25	16	8
PSA-146b	Ciprofloxacin	0.125	32	16
	Levofloxacin	0.5	32	8

Supplementary Figures

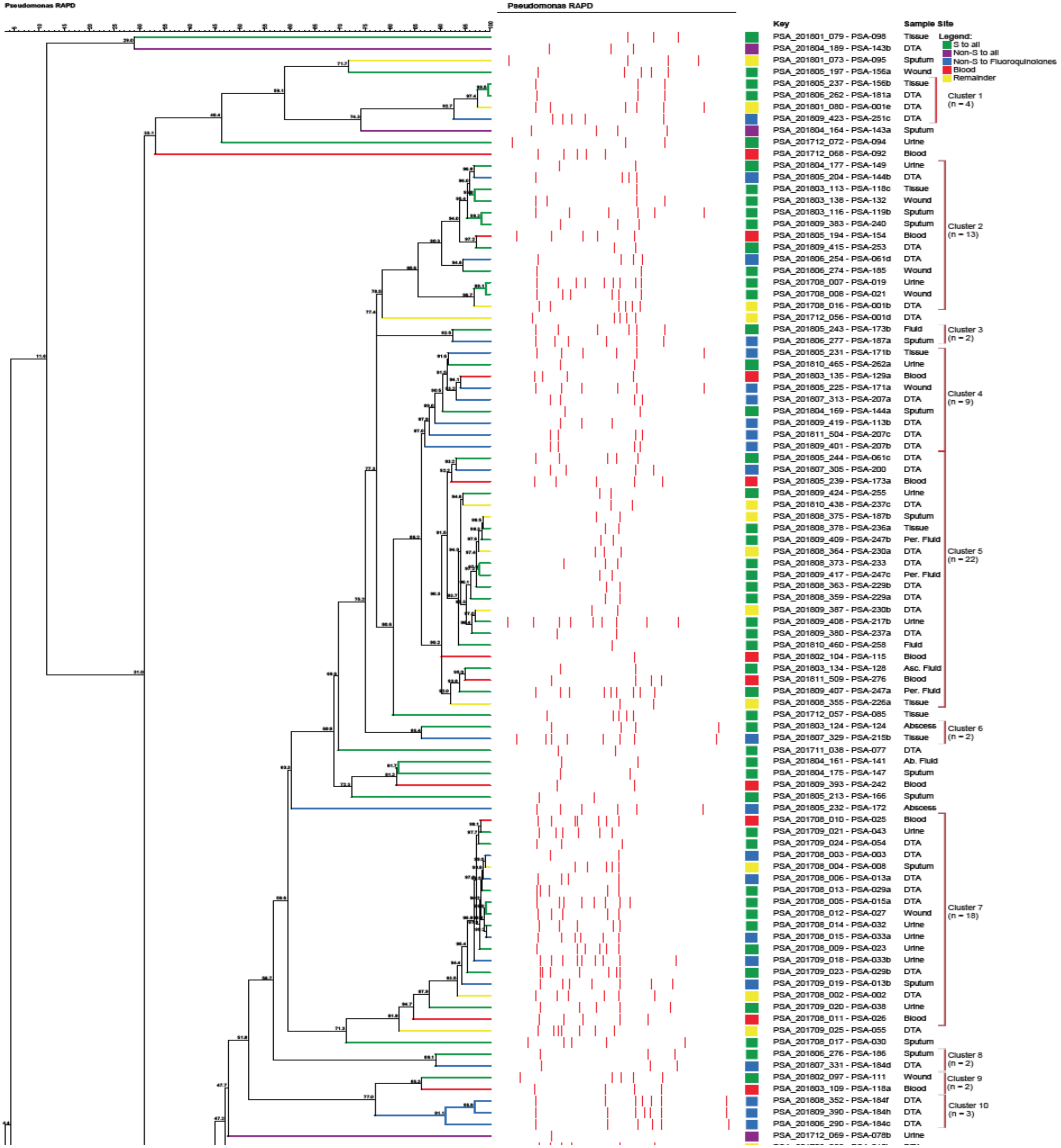

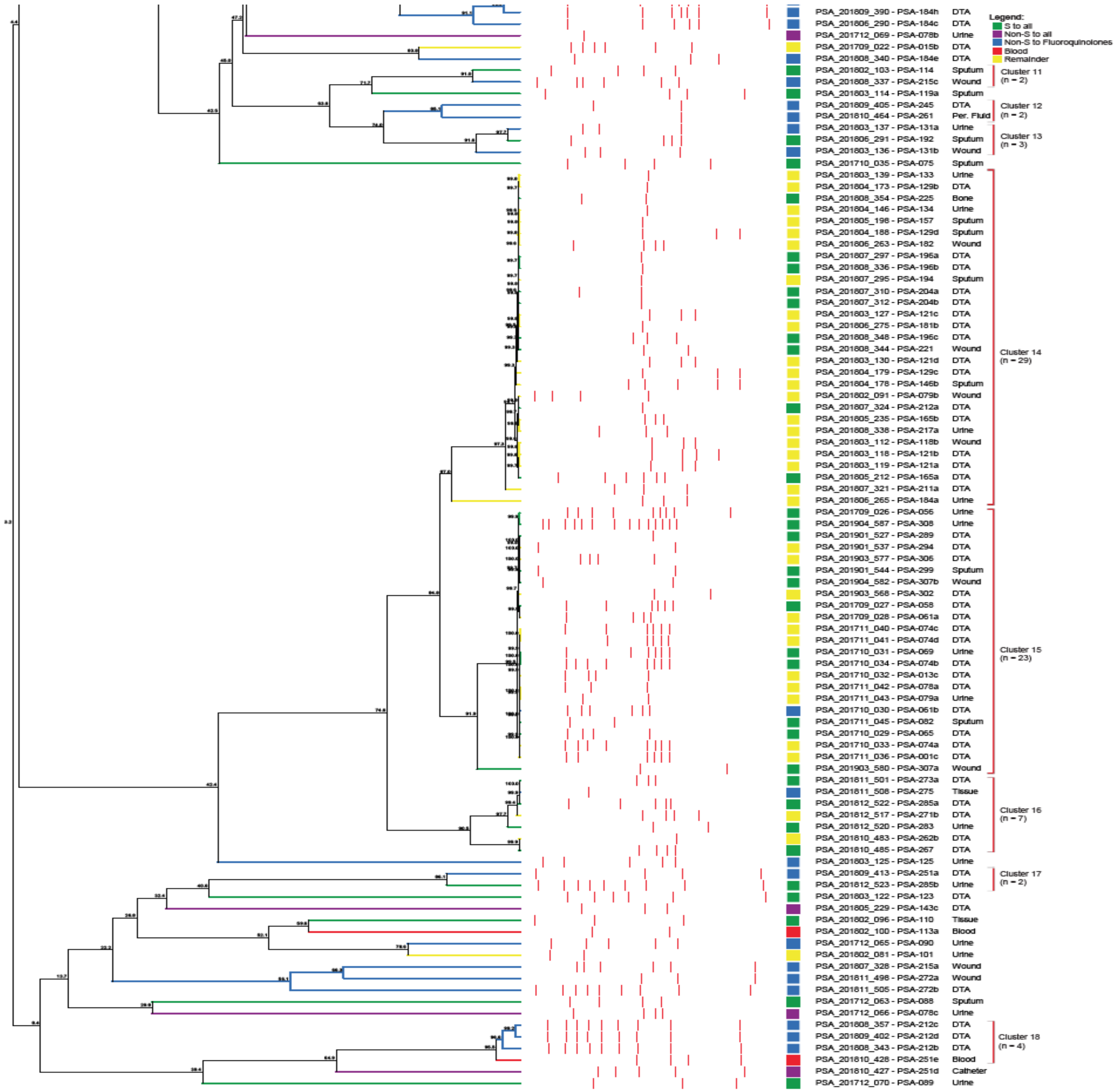

Figure S1: The UPGMA dendrogram of all isolates that were analyzed by RAPD.

Color coding indicates the group that each isolate belongs to. Red side brackets indicate clusters for similarity.

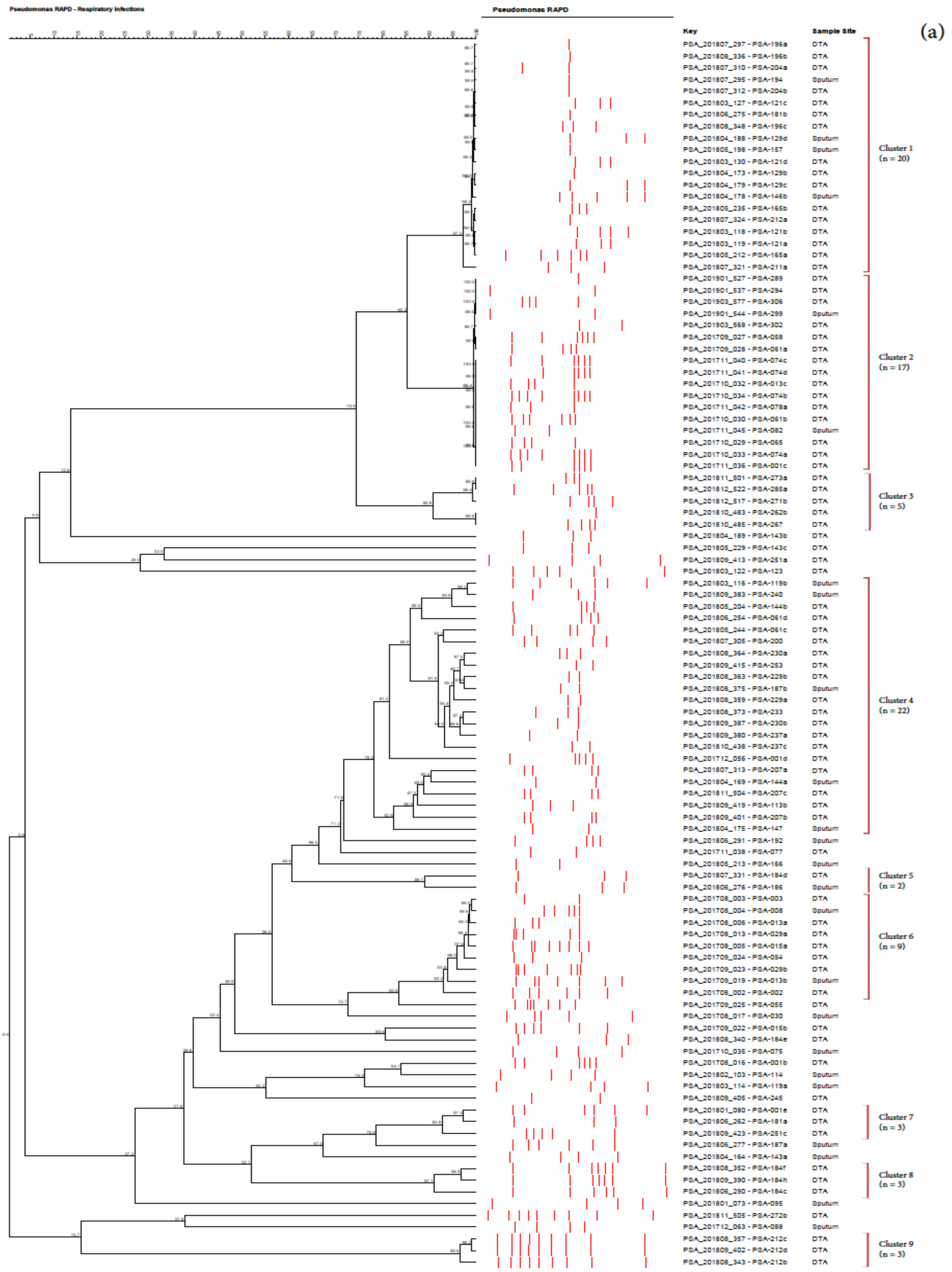

Figure S2: The UPGMA dendrogram of select isolates sampled from respiratory sites

Red side brackets indicate clusters of similarity.

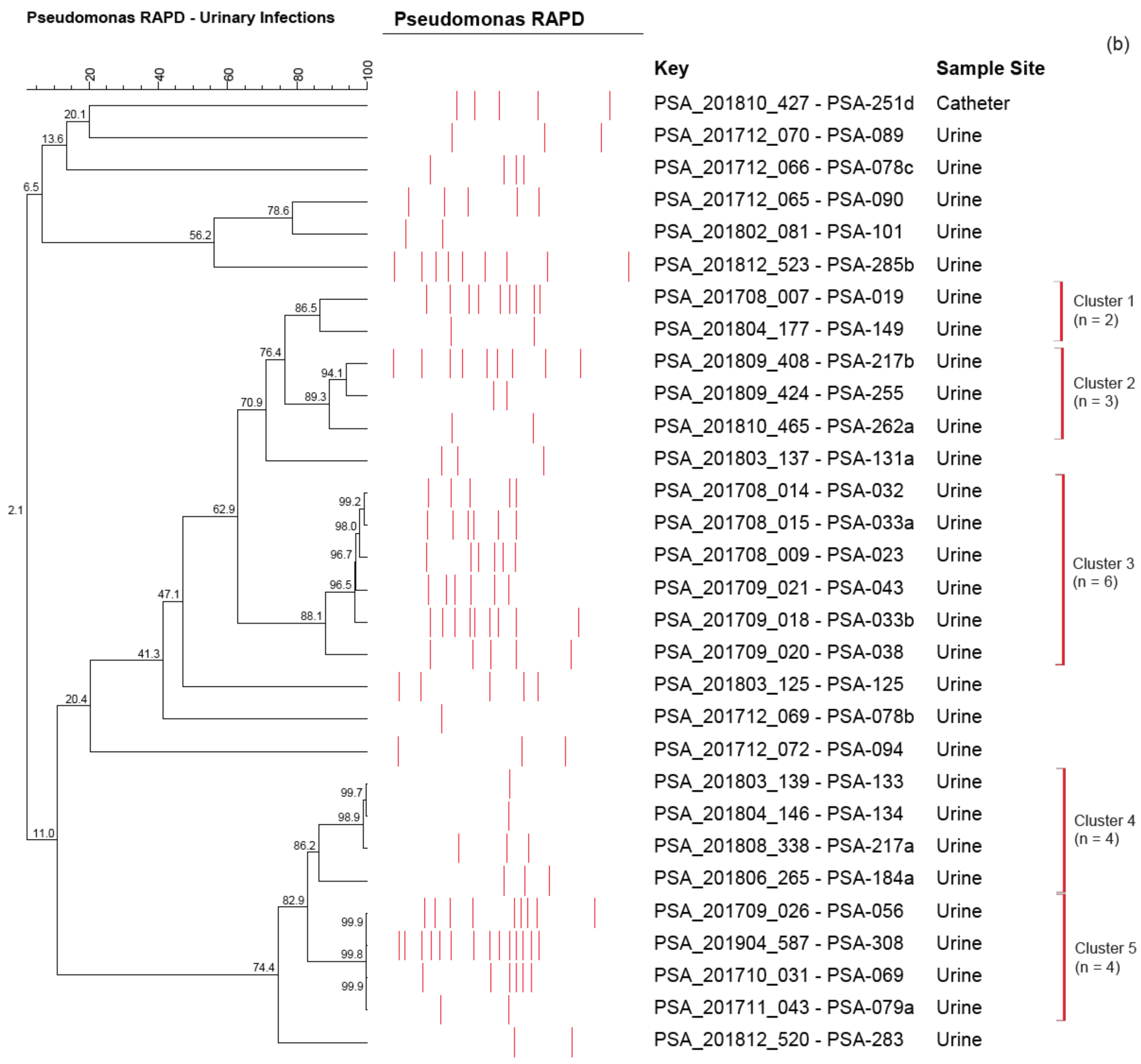

Figure S3: The UPGMA dendrogram of select isolates sampled from urine

Red side brackets indicate clusters of similarity.

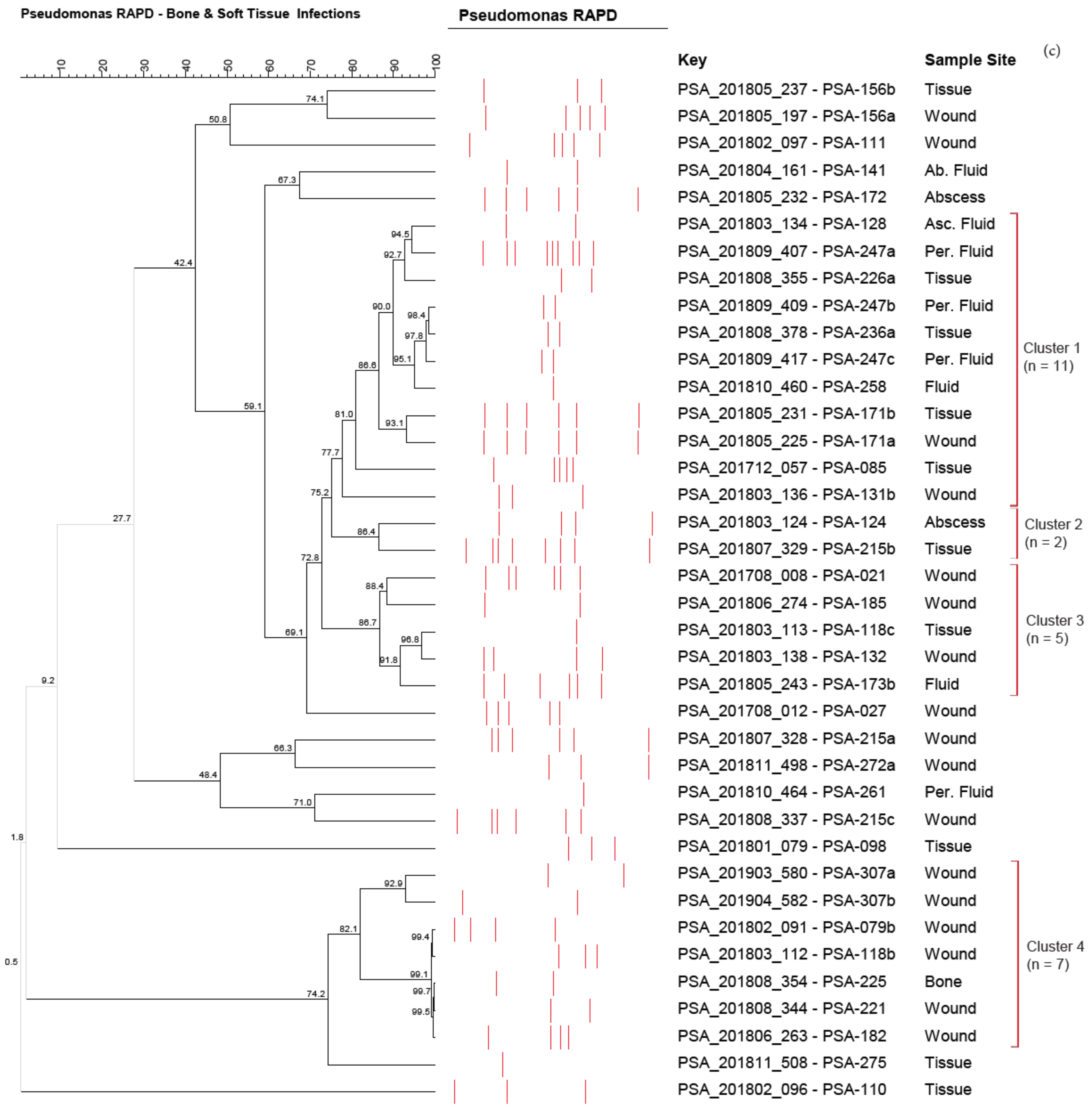

Figure S4: The UPGMA dendrogram of select isolates sampled from bone and soft tissue sites

Red side brackets indicate clusters of similarity.

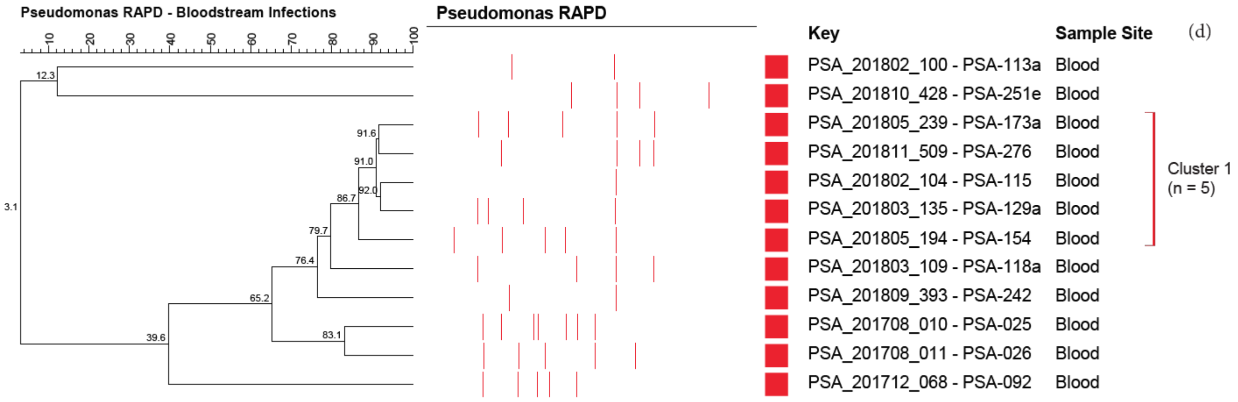

Figure S5: The UPGMA dendrogram of select isolates sampled from blood

Red side brackets indicate clusters of similarity.

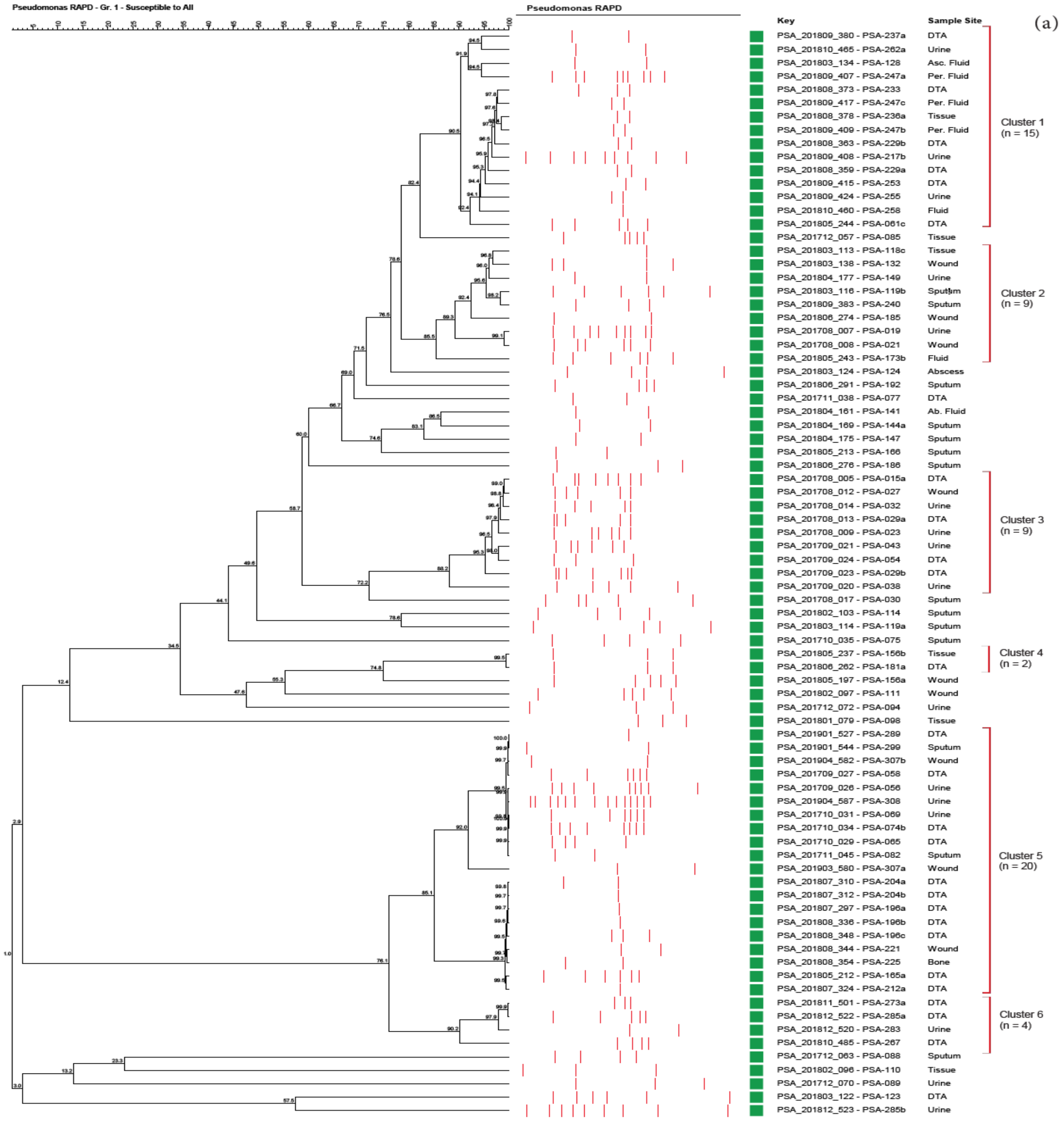

Figure S6: The UPGMA dendrogram of select isolates that are susceptible to all tested antibiotics.

Red side brackets indicate clusters of similarity

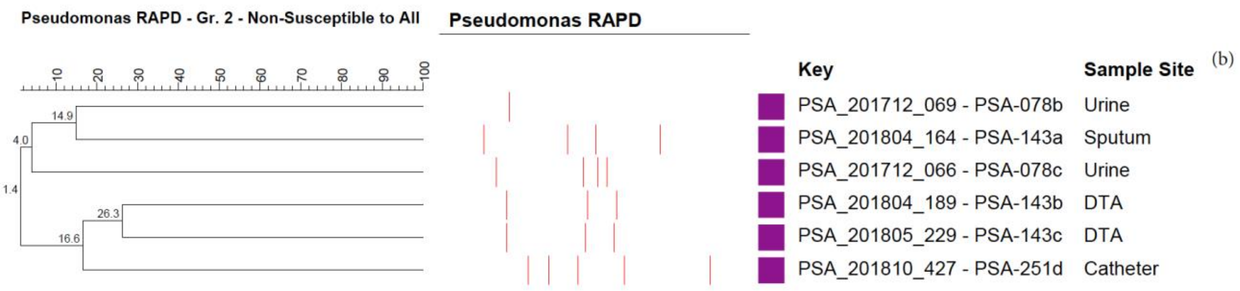

Figure S7: The UPGMA dendrogram of select isolates that are non-susceptible to all tested antibiotics.

Red side brackets indicate clusters of similarity

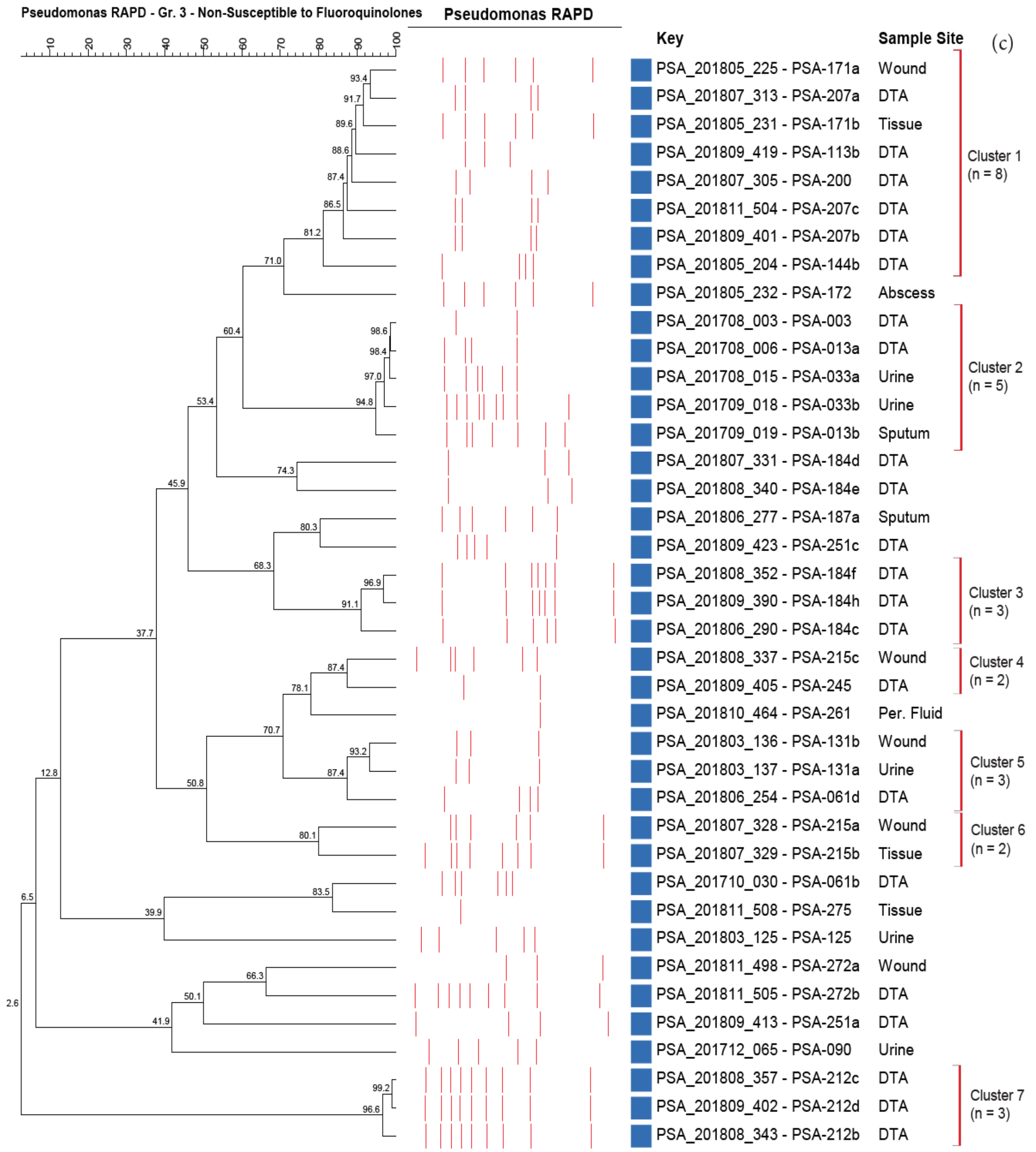

Figure S8: The UPGMA dendrogram of select isolates that are non-susceptible to fluoroquinolones. Red side brackets indicate clusters of similarity

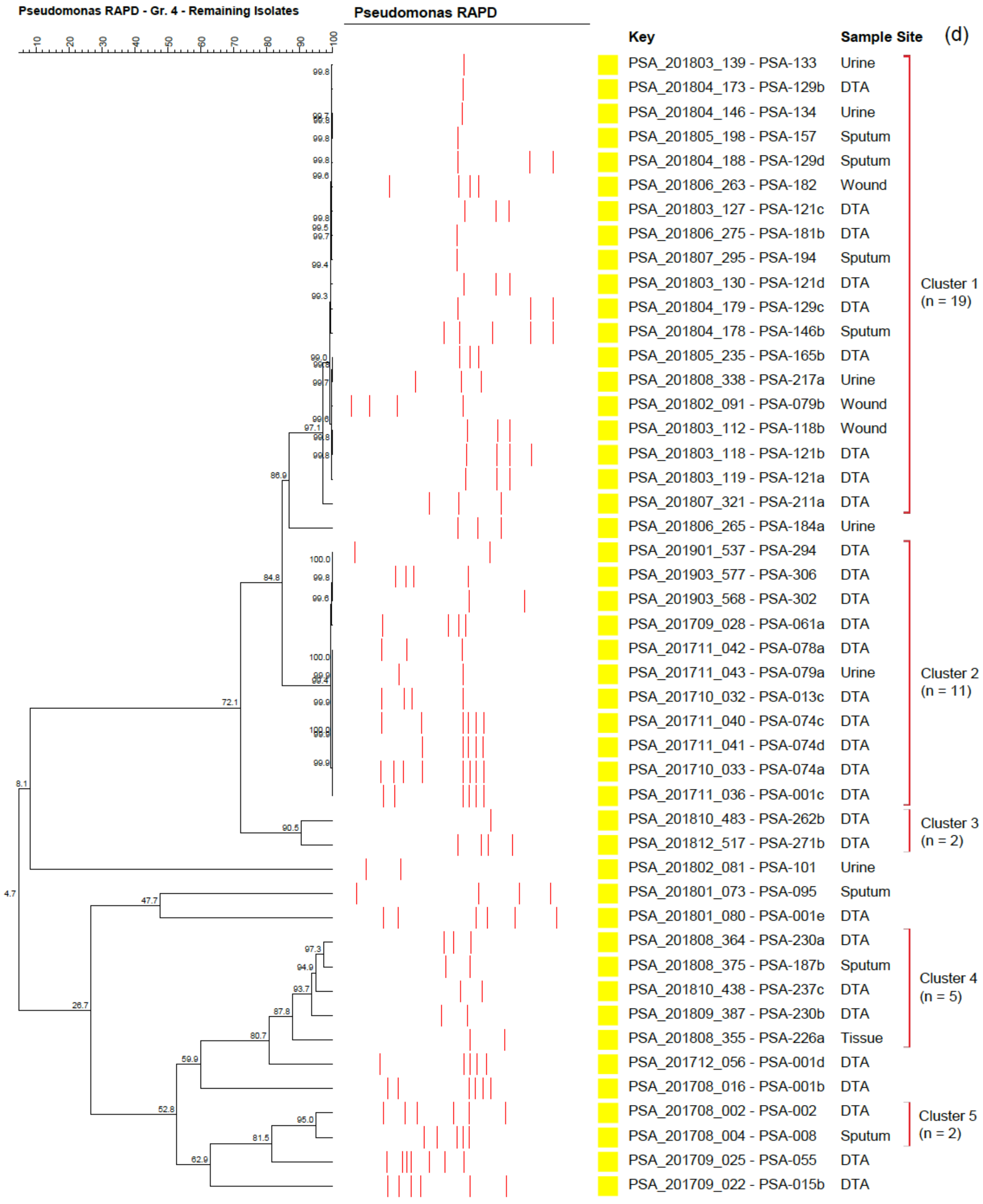

Figure S9: The UPGMA dendrogram of select isolates that are grouped as remaining isolates

Red side brackets indicate clusters of similarity

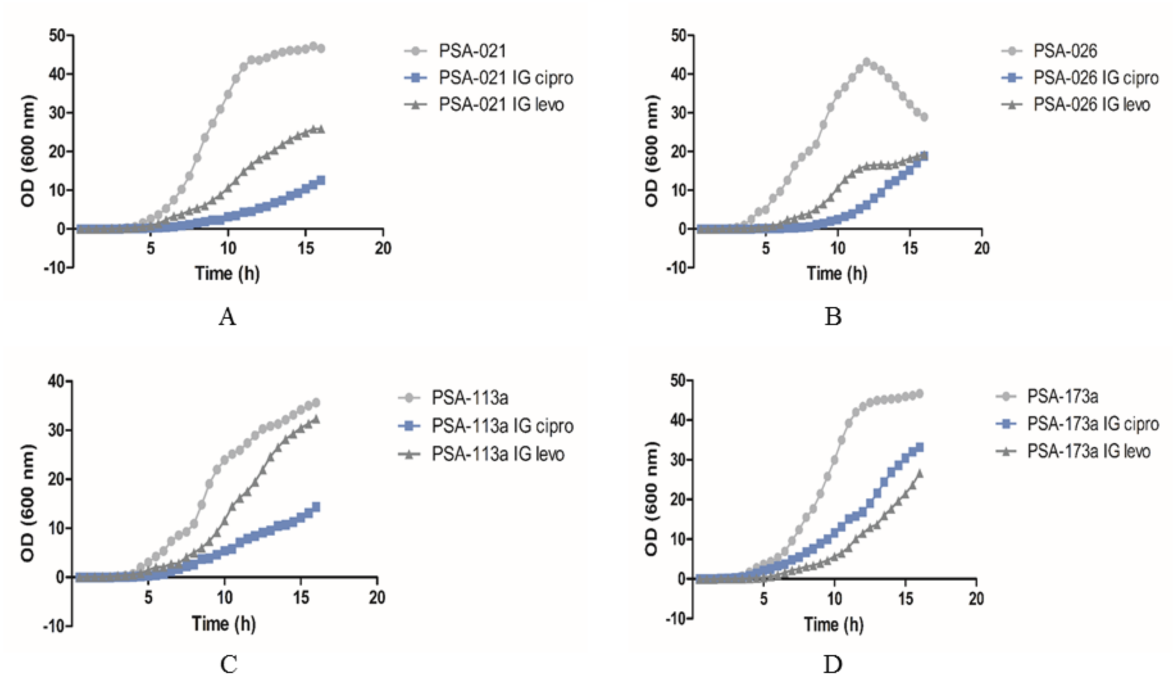

Figure S10: Fitness cost assay results of isolates PSA-021, PSA-026, PSA-113a and PSA-173a compared to their induced mutants

IG cipro: induced against ciprofloxacin; IG levo: induced against levofloxacin

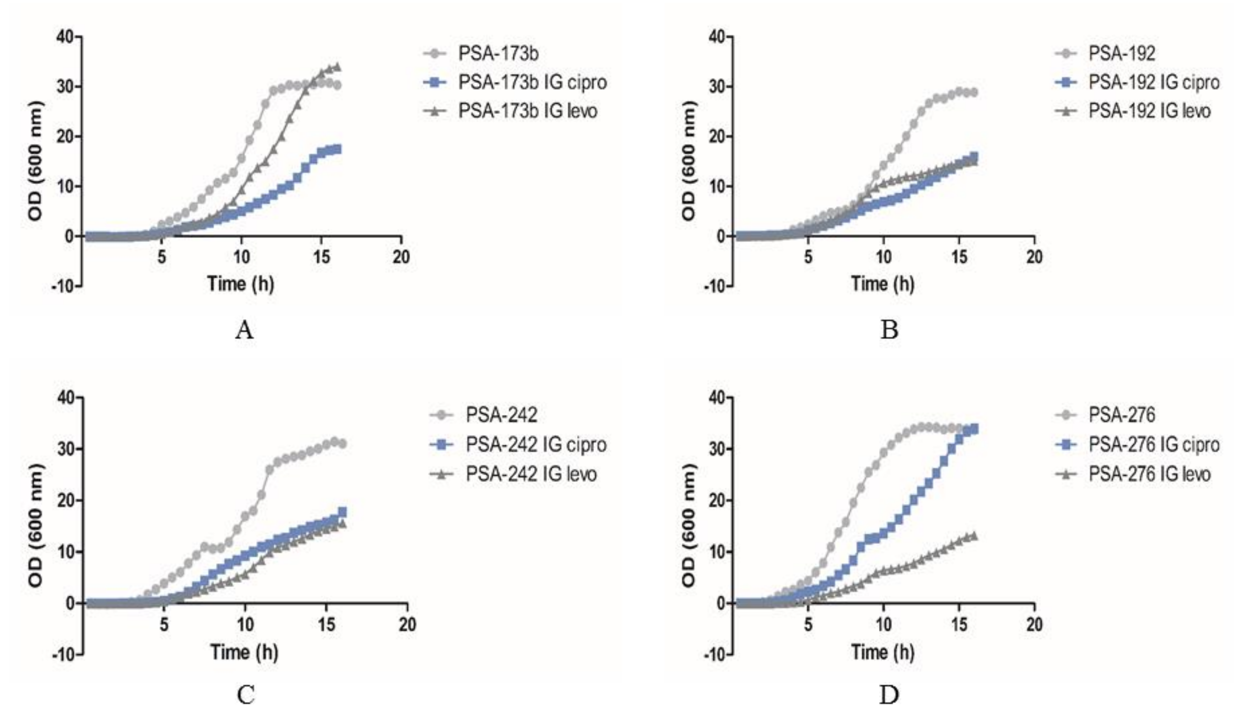

Figure S11: Fitness cost assay results of isolates PSA-173b, PSA-192, PSA-242 and PSA-276 compared to their induced mutants

IG cipro: induced against ciprofloxacin; IG levo: induced against levofloxacin

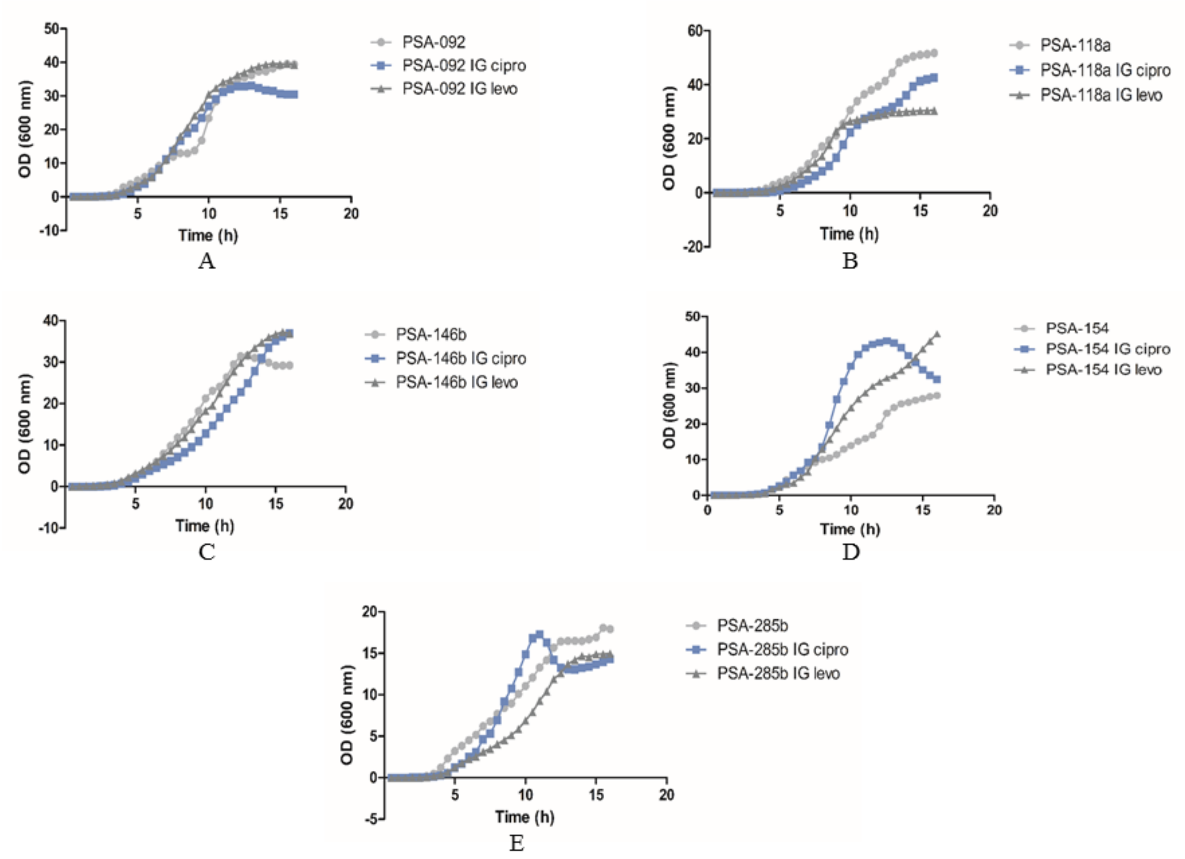

Figure S12: Fitness cost assay results of isolates PSA-092, PSA-118a, PSA-146b, PSA-154 and PSA-285b compared to their induced mutants

IG cipro: induced against ciprofloxacin; IG levo: induced against levofloxacin

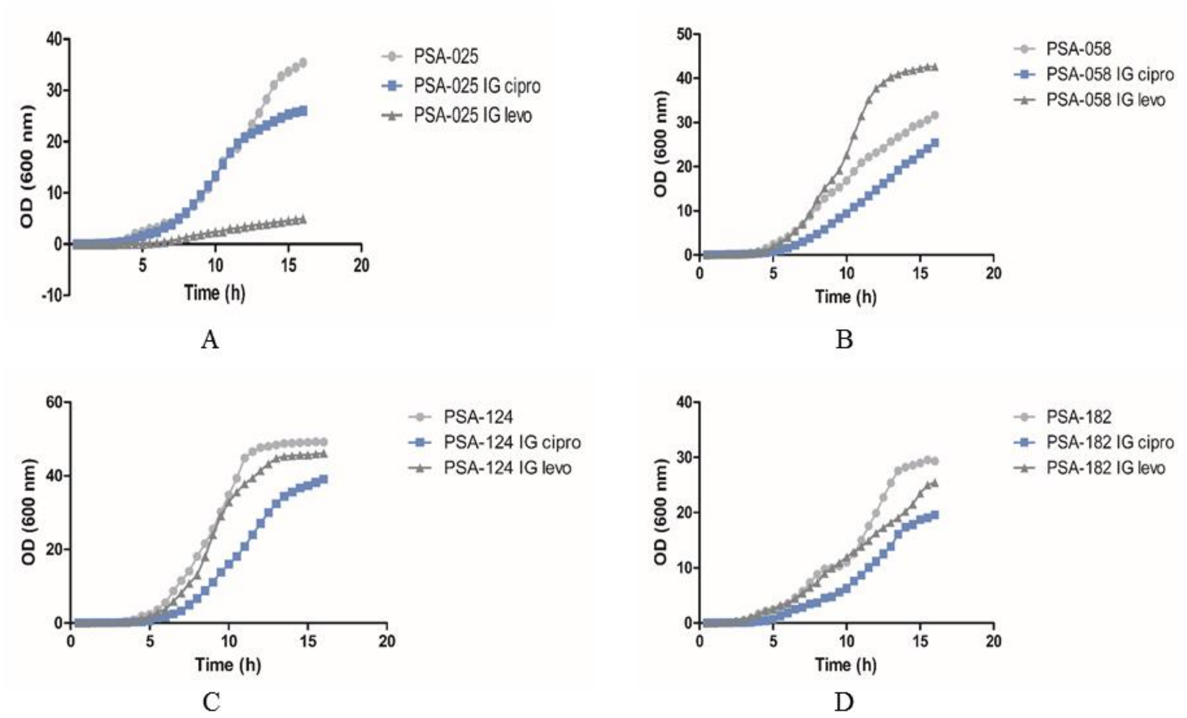

Figure S13: Fitness cost assay results of isolates PSA-025, PSA-058, PSA-124, and PSA-182 compared to their induced mutants

IG cipro: induced against ciprofloxacin; IG levo: induced against levofloxacin
